# Psychosocial factors associated with physical activity and sedentary behavior in adolescents: evidence from physical education contexts in Quito

**DOI:** 10.3389/fpsyg.2026.1813419

**Published:** 2026-04-22

**Authors:** David Pérez-Jorge, Edison Rodrigo Arias-Moreno, Ricardo De La Vega Marcos, Ángel Freddy Rodríguez-Torres, Miriam Catalina González-Afonso

**Affiliations:** 1 Department of Teaching and Educational Research, University of La Laguna, La Laguna, Spain; 2 Libertad Institute of Technology, Quito, Ecuador; 3 Department of Physical Education, Sports, and Human Movement, Autonomous University of Madrid, Madrid, Spain; 4 Faculty of Physical Culture, Central University of Ecuador, Quito, Ecuador

**Keywords:** autonomy support, physical activity, physical education, screen time, self-determination theory, self-efficacy

## Abstract

Physical Education (PE), as a psychoeducational context, plays a central role in understanding adolescents’ engagement in physical activity and sedentary behavior. This study aimed to examine the associations between psychosocial variables related to the PE experience and levels of physical activity both within PE and during leisure time, as well as recreational screen time among adolescents in Quito, Ecuador. A cross-sectional sequential explanatory mixed-methods design was employed, integrating quantitative and qualitative evidence. The quantitative sample included 5,515 students aged 12–18 years from 11 public, publicly funded private, and private schools in urban and rural areas. The qualitative component consisted of six student focus groups and one teacher focus group. Measures included physical activity, screen time, motivational orientation, situational intrinsic motivation in PE, self-esteem, motor self-efficacy, and perceived autonomy support. Analyses comprised descriptive statistics, sex-based comparisons, and Spearman correlations, complemented by thematic analysis of qualitative data. Results indicated that 29% of adolescents did not comply with the 2-h daily threshold for recreational screen time (95% CI: 27.80–30.20), with non-compliance increasing with age and being more prevalent among girls than boys. Bivariate associations revealed a small positive relationship between self-efficacy and leisure-time physical activity (rho = 0.050), whereas situational intrinsic motivation in PE showed a small negative association. Qualitative findings confirmed and extended these patterns, highlighting that screen time competes with academic demands and leisure, and that perceived competence and pedagogical climate are key factors shaping motivation and participation. Overall, the findings underscore the relevance of psychosocial factors within PE, while emphasizing the role of contextual and structural conditions in shaping adolescents’ physical activity and sedentary behavior. These results support the need for gender-sensitive and context-responsive school-based interventions to promote active lifestyles.

## 1 Introduction

Adolescence is a critical stage for consolidating habits that tend to persist into adulthood. During this period, a progressive decline in physical activity and an increase in time devoted to sedentary behaviors are commonly observed, particularly those linked to screen use (Da Penha Freire Silva et al., 2025; Haas et al., 2021). It is also important to note that screen time accounts for only a portion of total sedentary behavior, and that different screen-based activities may vary in terms of context, purpose and level of cognitive engagement, which may help to explain why the associations are sometimes modest or inconsistent across different studies (Zou et al., 2024; Zhang et al., 2025). From a public health perspective, this combination is concerning because it is associated with cardiometabolic conditions, mental health, and overall wellbeing (Bengtsson et al., 2025; Fu et al., 2025; Lundgren et al., 2025; Singh et al., 2026). Within a 24-h movement behavior perspective, these behaviors are best understood as interdependent components that coexist alongside sleep and share a finite daily time budget (Lu et al., 2021).

Beyond the immediate effects on physiological indicators, the literature has emphasized that activity and sedentary patterns acquired during adolescence show moderate stability across the life course, reinforcing the preventive relevance of this period from a life-course approach perspective.

International recommendations state that children and adolescents should engage, on average, in at least 60 min per day of moderate-to-vigorous physical activity, primarily aerobic in nature, and incorporate vigorous-intensity activities as well as muscle- and bone-strengthening activities at least 3 days per week (Bull et al., 2020; World Health Organization, 2020). Nonetheless, global evidence shows high prevalence rates of inactivity among adolescents, with consistent patterns of inequality by sex and context (Guthold et al., 2020). In Ecuador, available population-level data also indicate a relevant proportion of adolescents with insufficient physical activity and considerable levels of physical inactivity, with differences by age and area of residence (Instituto Nacional de Estadística y Censos, 2024).

However, available analyses in the country have been predominantly descriptive and have shown limited integration of school-based psychosocial variables, limiting understanding of the mechanisms underlying active behavior in specific educational contexts. This gap is relevant because adolescent-focused evidence consistently highlights perceived competence, self-efficacy, and perceived social support as key correlates and plausible pathway variables for sustained physical activity, often linking social resources and supportive environments to adolescents’ participation (Ren et al., 2020; Lubans et al., 2008; Babic et al., 2014). In school contexts, beliefs about competence and perceived support can shape students’ willingness to participate and to continue being active beyond the classroom (Lubans et al., 2011).

Taken together, this evidence highlights the need to identify factors associated with active behavior and sedentary patterns in concrete settings, particularly in urban environments such as Quito, where diverse educational and socioeconomic realities coexist. Despite growing international evidence on psychosocial correlates of adolescent physical activity, there remains limited mixed-methods research in Latin American urban contexts, particularly within PE settings.

Sedentary behavior is not limited to “not engaging in sports,” but includes prolonged periods of low-energy-demand activities, primarily sitting or reclining. Among adolescents, a substantial portion of sedentary time is recreational screen time (television, video games, internet, and social media), a useful indicator given its everyday relevance and its association with health and psychosocial adjustment variables (Haug, 2024; Panjeti-Madan and Ranganathan, 2023). However, recreational screen time should be interpreted as an operational indicator rather than a comprehensive measure of sedentary behavior, since it captures only a subset of sedentary time and may be linked to outcomes differently depending on the type of screen-based activity and how it is experienced (Huang et al., 2020; Zou et al., 2024). Recent conceptual work further argues that sedentary behaviors differ in context, purpose, and cognitive engagement (for example, more mentally passive versus more mentally active screen-based behaviors), which may partly explain heterogeneous findings across studies (Zhang et al., 2025; Zou et al., 2024). In addition, associations may vary by screen type and may be sensitive to developmental stage and sex, reinforcing the need to avoid treating total screen time as a single uniform exposure (Wang et al., 2025).

For conceptual clarity, it is important to distinguish between key terms used throughout this study. Sedentary behavior is defined as any waking behavior characterized by low energy expenditure (≤1.5 METs) while in a sitting, reclining, or lying posture. Recreational screen time refers specifically to screen-based sedentary activities performed during leisure time, such as internet use, social media, television, or video games, and is considered an operational indicator of sedentary behavior rather than a comprehensive measure. Free physical activity is defined as non-organized, self-directed physical activity performed outside structured or supervised contexts, typically during leisure time. These distinctions are relevant given ongoing debates in the literature regarding the conceptualization and measurement of sedentary behavior and physical activity patterns.

Recent studies have found associations between greater screen time and poorer mental health, including an increased risk of depressive symptoms, although the magnitude of the effect varies depending on study design, measurement approach, and type of digital use (Gao and Gao, 2024; Santos et al., 2023; Rodríguez-Torres et al., 2020). Prospective and meta-analytic evidence in adolescents also suggests that findings can differ across sedentary indicators and screen modalities, consistent with the heterogeneity of screen-based sedentary behaviors (Huang et al., 2020; Zou et al., 2024; Zhang et al., 2025). This line of evidence is consistent with broader research showing that maladaptive behavioral patterns during adolescence, such as procrastination and dysregulated time management, can accumulate health-related risks over time (Pérez-Jorge et al., 2024a,b).

Furthermore, analyses combining measures of physical activity and sedentary behavior have shown dose–response relationships with depression indicators in children and adolescents (Lu et al., 2024). This supports the value of considering physical activity and screen-based sedentary behavior together when interpreting adolescent mental health correlates.

Nevertheless, international guidelines primarily emphasize reducing sedentary behavior, particularly recreational screen time, without establishing a universal threshold based on definitive evidence (Bull et al., 2020; Chaput et al., 2020). In research practice and in some national guidelines, a cut-off point of 120 min per day has been used as an operational reference for screening and surveillance, facilitating comparisons and the communication of results (Canadian Society for Exercise Physiology, 2016). The available evidence does not allow definitive causal inferences, especially when self-reported measures and cross-sectional designs are used; therefore, these associations must be interpreted with caution. Reverse causality and bidirectional pathways remain plausible, and residual confounding cannot be ruled out, which reinforces the need for cautious language when discussing potential implications (Gao et al., 2024).

Physical activity among adolescents is a complex and multicausal behavior. Its explanation involves the interplay of individual, interpersonal, and contextual factors, as reflected in socioecological models and classic reviews on the correlates of physical activity (Bauman et al., 2012). Within this broader framework, adolescent evidence suggests that self-efficacy and perceived competence are especially relevant because they can function as process variables linking contextual influences to sustained participation (Ren et al., 2020; Lubans et al., 2008; Babic et al., 2014).

These models propose that active behavior does not depend exclusively on the availability of infrastructure or access to opportunities, but also on psychological processes mediated by school experiences and the subjective interpretation of the environment, particularly within structured health promotion initiatives implemented in school settings (Pérez-Jorge et al., 2021). In this regard, perceived support from peers and teachers has been linked to adolescents’ physical activity partly through its association with self-efficacy and competence-related beliefs (Ren et al., 2020; Lubans et al., 2011).

Within this framework, school Physical Education (PE) holds strategic value: it not only provides practice opportunities, but also shapes experiences, meanings, and motivational dispositions that may facilitate or hinder adherence to an active lifestyle beyond the classroom (Polet et al., 2021; Pérez-Jorge et al., 2021; Standage et al., 2003). This is consistent with evidence indicating that perceived competence and self-efficacy are closely tied to adolescents’ engagement in physical activity, supporting their relevance as targets within PE climates that aim to promote long-term participation (Babic et al., 2014; Lubans et al., 2008).

The transition from the curricular context to autonomous practice during leisure time has been identified as a major challenge in contemporary PE, particularly in educational systems with limited weekly instructional time. Mediator-focused research in youth suggests that strengthening self-efficacy and competence-related beliefs may be central for supporting this transfer from structured settings to self-directed activity outside school (Lubans et al., 2008).

The PE class functions as a microcontext with specific rules, pedagogical climate, and social relationships. When this climate promotes perceived competence, enjoyment, and participation, the likelihood of student engagement and transfer to leisure-time physical activity increases (Guan et al., 2023; Haas et al., 2021). Supportive interpersonal experiences may also reinforce adolescents’ confidence to be active and contribute to more sustained activity patterns beyond PE (Ren et al., 2020; Lubans et al., 2011). In contrast, when experiences of frustration, negative social comparison, or a lack of meaning predominate, students are more likely to disengage from practice, especially during transitions to higher grade levels.

Among psychosocial determinants, Self-Determination Theory provides a robust framework for understanding why students engage in or disengage from physical activity (Deci and Ryan, 2000; Ryan and Deci, 2017). From this perspective, motivational quality (rather than the quantity of motivation) is key to explaining behavioral persistence and the internalization of active habits. This theory posits that the quality of motivation largely depends on the satisfaction of basic psychological needs (autonomy, competence, and relatedness). Adolescent-focused evidence also supports the practical relevance of competence-related beliefs and self-efficacy as process variables linked to sustained participation, particularly in school-based contexts (Ren et al., 2020; Lubans et al., 2008; Babic et al., 2014). In PE, when teachers support autonomy, provide informative feedback, and create opportunities for choice and meaningful participation, intrinsic motivation and engagement tend to increase (Kruse et al., 2024; Langøy et al., 2024). Evidence also suggests that autonomy support is directly or indirectly linked to higher levels of physical activity, mediated by psychological needs and more self-determined forms of motivation (Wanwan and Khairani, 2025).

In addition to Self-Determination Theory, Achievement Goal Theory provides a complementary perspective for understanding motivation in Physical Education contexts. While SDT focuses on the quality of motivation and the satisfaction of basic psychological needs, Achievement Goal Theory emphasizes how individuals define competence and success, distinguishing between task (mastery) and ego (performance) orientations. In the present study, both frameworks are considered complementary, as they capture different but interrelated dimensions of adolescent motivation in physical activity contexts.

Social relationships in the classroom represent another key dimension: the quality of the teacher–student relationship, as well as peer acceptance, is associated with interest, enjoyment, and engagement in PE, and may modulate participation in physical activity (Chen et al., 2025; De Bruijn and Grimminger-Seidensticker, 2025). Likewise, self-efficacy, defined as the belief in one’s ability to perform actions successfully, has been consistently linked to active behavior (Bandura, 1997; Lin et al., 2024) and more broadly to indicators of adolescent psychological adjustment and flourishing in educational contexts (Pirrone et al., 2025) and has been shown to mediate the relationship between social support, psychological needs, and active behavior, underscoring its role in school-based interventions (Li et al., 2024; Lin et al., 2024). In the present study, these variables are analyzed in associative rather than causal terms, given the cross-sectional design.

Complementarily, self-esteem has been associated with sports participation and indicators of adjustment and may contribute to a sense of personal worth that sustains persistence in practice, especially when supported by experiences of competence and belonging (Moral-García et al., 2021).

International evidence consistently shows sex differences in physical activity levels, with lower participation in moderate-to-vigorous intensity among girls and greater exposure to sedentary behavior in certain subgroups, although profiles vary by cultural and school contexts (Guthold et al., 2020; Haug, 2024). In PE, differences by sex and educational stage have also been reported in motivation, satisfaction of psychological needs, and enjoyment—variables closely linked to engagement and continuity of practice (Navarro-Patón et al., 2024).

The literature also suggests that sex differences may be mediated by social norms, cultural expectations, and differential structural opportunities, beyond individual dispositions, reinforcing the need for disaggregated analyses.

In addition to sex, the institutional context may shape relevant inequalities: available resources, infrastructure, academic workload, opportunities for organized practice, and teaching styles may vary across schools with different governance structures and between urban and rural areas. In Ecuador, social surveillance data indicate differences across sociodemographic profiles in insufficient physical activity and sedentary behavior, suggesting that analyses should consider the environment as a substantive, not merely descriptive, component (Instituto Nacional de Estadística y Censos, 2024).

Although quantitative studies allow for the estimation of prevalences and associations, qualitative data provide indispensable information for interpreting underlying mechanisms and contextual conditions. In PE, where students’ and teachers’ perceptions of the pedagogical climate, barriers, supports, and school culture are decisive, a mixed-methods approach facilitates the integration of the “what happens” with the “why it happens,” and helps guide more realistic and culturally responsive interventions. This rationale is particularly relevant in settings with limited local evidence and high institutional heterogeneity, such as the Quito educational system. In addition, given that recreational screen time is an operational proxy and screen-based sedentary behaviors can differ in context and cognitive engagement, qualitative evidence can help interpret heterogeneous or small associations that may otherwise appear inconsistent (Zou et al., 2024; Zhang et al., 2025). The integration of quantitative and qualitative data enables not only the description of patterns but also the exploration of contextual explanations for weak or counterintuitive associations.

Overall, the evidence suggests that active behavior and sedentary lifestyles in adolescents should be analyzed from an integrated perspective that combines individual, school-based, and contextual determinants, particularly in urban environments characterized by high institutional heterogeneity. Consistent with 24-h movement behavior perspectives, we interpret physical activity and screen-based sedentary behavior as part of interconnected daily behavior patterns, while maintaining cautious, non-causal language given the cross-sectional design (Lu et al., 2021).

Based on this context, the present study aimed to:

- Describe levels of physical activity (in PE classes, organized physical activity, and free physical activity) and sedentary behavior (recreational screen time), according to sex, age, school type, and area (urban or rural).
- Analyze the associations between psychosocial factors (motivational orientation, situational intrinsic motivation in PE, self-esteem, self-efficacy, and perceived autonomy support) and physical activity, as well as screen time.
- Examine differences in physical activity, sedentary behavior, and psychosocial variables according to sex and school type.
- Explore, through qualitative evidence, students’ and teachers’ perceptions of motives, barriers, and pedagogical conditions related to physical activity practice and screen use, in order to contextualize and interpret the quantitative findings.

## 2 Methodology

A mixed-methods, cross-sectional study with a sequential explanatory design was developed: in the first phase, quantitative evidence was collected and analysed, and subsequently, the qualitative component was conducted to deepen the interpretation of the observed patterns (Creswell and Plano Clark, 2018). The integration between phases was achieved through connection (the quantitative results guided the script and sampling of the qualitative component) and interpretation (the qualitative findings were used to contextualise and explain the differences and associations identified in the quantitative phase) (Fetters et al., 2013).

Additionally, joint displays were constructed to systematically integrate quantitative and qualitative findings, allowing explicit comparison between statistical results and qualitative themes, in line with best practices in mixed-methods research (Fetters et al., 2013).

The mixed design responded to a complementary logic: while the quantitative phase enabled estimation of prevalences and associations in a large sample, the qualitative phase provided interpretive depth to understand the contextual mechanisms underlying the observed statistical patterns.

### 2.1 Participants and sampling

#### 2.1.1 Quantitative sample

A total of 5,515 students aged 12 to 18 years participated in the study. They were enrolled in Educación General Básica Superior (8°, 9° y 10°) and Bachillerato (1°, 2° y 3°) in the city of Quito (Ecuador). Eleven schools were included from urban and rural areas, with different school types: fiscal (public), fiscomisional (privately managed with partial public funding), and private (particular). A non-probabilistic convenience sampling strategy was used, conditioned by accessibility and institutional authorization. In total, 3,594 students attended urban schools and 1,921 attended rural schools; by school type, 2,498 attended public schools, 1,533 attended fiscomisional schools, and 1,484 attended private schools. The sex distribution was 3,199 boys (58.0%) and 2,316 girls (42.0%). Quantitative data were collected between May 23 and July 29, 2022.

The large sample size allowed for estimates with high statistical precision; nonetheless, given the non-probabilistic convenience sampling, the results should be interpreted as associations within the sample rather than as strictly representative estimates of the total adolescent population of Quito.

Given the large sample size, the study had high statistical power to detect small effect sizes. For example, correlations of approximately rho = 0.05 can be detected with high statistical power (typically > 0.90), indicating that the analyses were adequately powered for the observed effects.

Inclusion criteria and data quality. Enrolled students who were present on the day of questionnaire administration and who provided informed consent were included. For each analysis, the effective sample size was reported when key variables were missing.

The percentage of missing data was below 5% for the main variables; analyses were conducted using valid cases per variable (pairwise deletion), given the low proportion of non-response.

#### 2.1.2 Qualitative sample

Purposive sampling was used to maximize heterogeneity across educational stages and school profiles.

The selection followed a maximum-variation sampling criterion to capture a diversity of experiences across educational stages and school type.

Six focus groups were conducted with students (three from EGBS and three from Bachillerato), with 7 to 10 participants per group and representation of both sexes. In addition, one focus group was conducted with PE teachers, organized into two subgroups to facilitate interaction. The sessions took place in February 2023 and were audio-recorded.

Group size was determined in accordance with common methodological recommendations (6–10 participants) to promote interaction and depth of discussion.

### 2.2 Variables and instruments

The variables were grouped into behavioral, psychosocial, and sociodemographic variables.

#### 2.2.1 Behavioral variables

- Physical activity. It was assessed using a 7-day recall based on the International Physical Activity Questionnaire, adolescent version (IPAQ-A), estimating minutes of at least moderate-intensity activity, differentiated by domains: PE classes (Monday to Friday); organized physical activity (with supervision); and free physical activity (without supervision) (Hagströmer et al., 2008). Minutes were counted when the activity lasted at least 10 consecutive minutes. The IPAQ-A has shown acceptable test–retest reliability in adolescent populations, although the potential overestimation inherent in self-reported measures is acknowledged.

For data processing, continuous indicators were generated: weekly minutes per domain (PE classes, organized physical activity, and free physical activity) and a weekly total (sum of the three domains). These indicators were used to describe practice levels and to conduct association analyses with psychosocial variables.

Additionally, the percentages of students meeting the international recommendation of 60 min per day were explored descriptively as a contextual reference.

- Sedentary behavior, screen time. It was assessed using the Adolescent Sedentary Activity Questionnaire (ASAQ), differentiating between weekdays and weekends for television or videos, video games, internet and social media use, and homework with or without a computer (Hardy et al., 2007), recording daily hours and minutes of screen use on weekdays and weekends for television, video games, internet browsing and social media, and homework with or without a computer. Time values were weighted to calculate an average total daily value, accounting for both school days and weekends.

Subsequently, time values were categorized according to an operational threshold of 120 min per day (meets or does not meet) (Tremblay et al., 2016).

The 120-min cut-off is widely used as an epidemiological reference in surveillance studies and does not imply a definitive clinical threshold.

#### 2.2.2 Psychosocial variables

- Motivational orientation (Achievement Goal Theory): assessed using the Task and Ego Orientation in Sport Questionnaire (TEOSQ; Ruiz-Juan et al., 2011), which captures task (mastery) and ego (performance) goal orientations related to competence evaluation.
- Situational intrinsic motivation in PE (Self-Determination Theory): assessed using the intrinsic motivation subscale of the Situational Motivation Scale (SIMS) (4 items; 7-point scale) (Burgueño et al., 2018; Guay et al., 2000; Martín-Albo et al., 2009), which captures the quality of motivation along the autonomy continuum.
- Self-esteem: Measure based on Coopersmith (range 0 to 100), categorized as low, moderate, and high (Coopersmith, 1981).
- Motor self-efficacy: Motor Self-Efficacy Scale (10 items; 4-point scale; range 10 to 40) (Hernández-Álvarez et al., 2011).
- Autonomy support: Perception Scale of Support for Autonomy in PE Classes (EPAACEF) (34 items; 4-point scale), including a global score and subdimensions (Aguado-Gómez et al., 2017).

For all scales, internal consistency was calculated in the sample (Cronbach’s alpha or omega), and descriptive statistics were obtained (mean, standard deviation, and observed range).

#### 2.2.3 Sociodemographic variables

Gender, age, school type, and area (urban, rural).

#### 2.2.4 Qualitative component scripts

Semi-structured scripts were used for students and teachers, focusing on: (a) motives and barriers to practice; (b) experiences in PE; (c) use of screens and organisation of free time; and (d) perceived pedagogical conditions and materials. The script was adjusted based on preliminary quantitative results to explore plausible explanations for differences by gender, age, area, and ownership.

### 2.3 Procedure

A pilot test was conducted to refine the wording, the response format (hours and minutes), and the duration (15 to 38 min).

The pilot test improved the semantic clarity of certain items and optimized participants’ understanding of the time format in hours and minutes.

Following institutional authorization, written informed consent was obtained from legal guardians, and assent from students. The questionnaire was administered in person during school hours. To ensure anonymity, a numerical code and coding by school, area, and school type were assigned; efforts were made to include absent students on subsequent dates.

The focus group interviews were conducted on school premises. They were audio-recorded for transcription and analysis; video recording was avoided to minimize the risk of identification.

### 2.4 Data analysis

#### 2.4.1 Quantitative analysis

Differences in proportions were evaluated using chi-square tests, reporting *χ*^2^ values, degrees of freedom, exact *p*-values, and Cramér’s V as the effect size.

Associations between variables were estimated using Spearman’s rank correlation (rho), interpreting effect magnitude alongside statistical significance. In presenting the results, substantive interpretation (effect size and theoretical coherence) was prioritized over isolated statistical significance, given the large sample size.

Nonparametric correlations were used because several behavioral variables are not normally distributed. The magnitude of the coefficients was interpreted following conventional criteria (≈ 0.10 small, ≈ 0.30 moderate, ≥ 0.50 large).

#### 2.4.2 Qualitative analysis

The analysis was conducted using Atlas.ti (version 24) through thematic analysis (Braun and Clarke, 2006): verbatim transcription, coding by meaning units, and grouping into categories and subcategories. The coding combined a deductive approach (categories aligned with the objectives) and an inductive approach (emergent codes). To enhance the credibility of the analysis, the coding system was iteratively reviewed, and representative textual evidence was retained for each theme.

Two researchers reviewed the coding framework and discussed discrepancies until consensus was reached, thereby strengthening the credibility of the analysis. To enhance the trustworthiness of the qualitative analysis, several strategies were implemented. First, an iterative coding process was conducted, in which coding decisions and category development were continuously reviewed and refined, maintaining an audit trail of analytical decisions throughout the process.

Second, reflexivity was considered during analysis, acknowledging that the researchers’ backgrounds in physical education and educational research may have influenced data interpretation; this was addressed through ongoing discussion and critical reflection within the research team.

Third, attention was paid to identifying both convergent and less frequent or divergent perspectives within the data to avoid over-simplification and strengthen the credibility of the thematic interpretation.

Consistent with reflexive thematic analysis approaches, formal inter-rater reliability coefficients were not calculated; instead, analytical rigor was supported through collaborative coding, iterative discussion, and consensus-building.

Member-checking with participants was not conducted, which is acknowledged as a limitation of the qualitative component.

#### 2.4.3 Integration between phases

Integration was carried out at two levels: (1) connection, using quantitative results to guide purposive selection and the design of the interview guide; and (2) interpretation, contrasting convergences and divergences between quantitative patterns (sex, age, area, and school type) and qualitative explanations related to pedagogical climate, perceived competence, autonomy, resources, and time management. Integration was operationalized through comparison matrices that linked specific quantitative patterns to emerging qualitative categories.

### 2.5 Ethical considerations

The study was conducted using non-invasive and minimal-risk procedures, in accordance with the principles of the Declaration of Helsinki and the national educational regulations in force at the time of data collection. Participation was voluntary, without incentives or academic consequences. Institutional authorization was obtained from the participating schools, as well as informed consent from legal guardians and assent from the students, who were informed of their right to withdraw at any time.

No identifying data were collected, and the information was coded and analyzed in aggregated form exclusively for scientific purposes. In the qualitative component, sessions were audio-recorded only, with prior consent, and video recording was avoided to minimize any risk of identification.

According to the current Ecuadorian regulatory framework, educational studies based on anonymous surveys and minimal risk are not subject to mandatory review by the Comités de Ética en Investigación en Seres Humanos (CEISH), whose jurisdiction primarily focuses on biomedical research.

## 3 Results

The results are presented according to the study objectives. The quantitative component is reported using percentages and association coefficients, and the qualitative component is integrated to contextualise the patterns observed in PE students and teachers. Given the sample size, priority is given to the joint interpretation of effect size and theoretical consistency, avoiding causal inferences in line with the cross-sectional design.

### 3.1 Sample characteristics

A total of 5,515 school-aged adolescents aged 12 to 18 years from the city of Quito participated. Of the total, 58.0% were males (*n* = 3,199) and 42.0% were females (*n* = 2,316). Most belonged to urban centres (65.2%; *n* = 3,594) and public schools (45.3%; *n* = 2,498). Table 1 summarises these characteristics.

**Table 1:** Sociodemographic characteristics of the sample.

Variable	Category	*n*	%
Sex	Male	3.199	58.0
	Female	2.316	42.0
Zone	Urban	3.594	65.2
	Rural	1.921	34.8
School type	Fiscal	2.498	45.3
	Fiscomisional	1.533	27.8
	Private	1.484	26.9

### 3.2 Objective 1: levels of sedentary behavior (screen time) and physical activity

Across the sample as a whole, non-compliance with the two-hour daily threshold was particularly concentrated in recreational internet use (29% of the total) and increased with age, reaching higher values from age 16 onward (Table 2). Non-compliance was lower for homework (18.82%) and remained around 20% for television (20.84%) and video games (23.27%).

**Table 2:** Percentage of screen time non-compliance (more than 2 h per day) by age and type of activity.

Age	TV (does not comply)	Schoolwork (does not comply)	Games (does not comply)	Internet (does not comply)
12	19.71	20.57	20.00	15.29
13	20.20	17.21	23.39	21.71
14	19.39	15.64	26.45	28.00
15	20.78	18.55	24.53	30.45
16	25.55	20.00	23.09	39.45
17	23.45	22.99	23.45	35.63
18+	23.18	25.85	22.63	35.68
Total	20.84	18.82	23.27	29

Compared to males, females showed greater non-compliance in recreational internet use (35.86% versus 24.71%). In contrast, homework-related screen time was lower among males (16.29%) than among females (22.86%) (Table 3).

**Table 3:** Percentage of screen time non-compliance (more than 2 h per day) by activity and gender.

Activity	Male (does not comply)	Female (does not comply)	Total (does not comply)
TV	18.57	24.00	21.29
Schoolwork	16.29	22.86	19.57
Games	23.29	23.43	23.36
Internet	24.71	35.86	29.00

Age differences showed an increasing trend, particularly in recreational internet use, with a progressive rise from ages 14–15 and peak values from age 16 onwards. Although the differences were descriptive, the pattern suggests a consolidation of recreational internet use during middle and late adolescence.

Sex differences in recreational internet use were statistically significant [*χ*^2^(1) = 84.30, *p* < 0.001, Cramér’s V = 0.12], indicating a small-to-moderate effect size. Tables 4–6 show clear differences by school type in the distribution of students who exceeded 1 h per week of physical activity (PA), and further demonstrate that the pattern varies by domain (PE, unsupervised PA, and total PA).

**Table 4:** Distribution by school type according to exceeding 1 h per week of PA in PE.

School type	Does not perform >1 h of PE	Perform >1 h of PE	Total
Fiscal	35%	45%	45%
Fiscomisional	5%	28%	28%
Private	60%	27%	27%

**Table 5:** Distribution by school type according to exceeding 1 h per week of unsupervised PA.

School type	Do not perform for more than 1 h without supervision	Performs >1 h without supervision	Total
Fiscal	55%	39%	45%
Fiscomisional	24%	30%	28%
Private	21%	31%	27%

**Table 6:** Distribution by school type according to exceeding 1 h per week of total PA.

School type	Does not perform >1 h total	Perform >1 h total	Total
Fiscal	55%	42%	45%
Fiscomisional	28%	28%	28%
Private	17%	30%	27%

In PE (Table 4), within the group exceeding 1 h per week, the largest proportion was in fiscal schools (45%), followed by fiscomisional schools (28%) and private schools (27%). However, among students who did not exceed 1 h in PE, private school students were markedly overrepresented (60%), whereas fiscal schools (35%) and, especially, fiscomisional schools (5%) were underrepresented. This contrast suggests that school type is associated with a differential pattern of engagement in weekly participation in the subject.

For unsupervised physical activity (Table 5), the distribution was more balanced between fiscomisional and private schools in the group exceeding 1 h, with very similar percentages (30 and 31%, respectively). In contrast, fiscal schools accounted for 39%. In the group reporting less than 1 h of unsupervised physical activity, the distribution was again concentrated in fiscal schools (55%), followed by private schools (21%) and fiscomisional schools (24%). In this case, differences between school types were less pronounced than in PE, suggesting that independent practice outside supervised contexts may depend on factors other than those operating within school hours. In this case, differences between school types are less pronounced than in PE, suggesting that independent practice outside supervision may depend on factors other than those operating within school hours.

Finally, in terms of total physical activity (Table 6), the group exceeding 1 h per week consisted mainly of students from state schools (42%), followed by students from private schools (30%) and fiscomisional (28%). In contrast, in the group that does not exceed 1 h in total, students from fiscal schools predominate (55%), followed by fiscomisional schools (28%) and, in particular, private schools (17%).

It should be noted that the data presented in each table do not directly correspond to prevalences “within each school type”; however, they help establish an overall pattern suggesting that school type is related to participation in physical activities, particularly in the context of PE, where private schools appear overrepresented among students reporting less than 1 h per week.

Associations between school type and exceeding 1 h per week were statistically significant [*χ*^2^(2) = 112.47, *p* < 0.001, Cramér’s V = 0.14], indicating a small-to-moderate effect size.

Given that the tables reflect the internal composition of the groups rather than adjusted prevalences within each school type, these results should be considered descriptive and exploratory.

### 3.3 Objective 2: relationship between psychosocial variables and physical activity

Spearman’s correlations showed statistically significant associations between free physical activity and psychosocial variables; however, the magnitudes were small (rho between 0.05 and 0.13), indicating that, although consistent, these relationships explain only a limited proportion of behavioral variability (Table 7).

**Table 7:** Spearman correlations (rho) between free physical activity and psychosocial variables, by gender.

Psychosocial variable	Free activity male (rho)	Free activity female (rho)	*p*
Self-efficacy	0.050	0.050	< 0.01
Intrinsic motivation	−0.123	−0.132	< 0.01
Motivational guidance	−0.060	0.077	< 0.01

The rho coefficients and overall significance (p) are reported.

In practical terms, coefficients in this range correspond to explained variances of less than 2%, reinforcing the need to interpret these links as contributing factors within a broader multifactorial framework.

Given the cross-sectional nature of the study, reverse causality cannot be excluded, and unmeasured variables (e.g., academic workload, access to facilities, or family norms) may also be involved. Therefore, caution is warranted when interpreting the sign of the coefficient.

### 3.4 Objective 3: differences in psychosocial profile by gender and school type

Motor self-efficacy differed by gender. The high self-efficacy category was more common among boys (53%) than among girls (41%), while girls were more concentrated at medium and low levels (Table 8). By school type, the differences were moderate, and the percentages remained within similar ranges (Table 9).

**Table 8:** Percentage of motor self-efficacy by gender.

Sex	Low self-efficacy	Average self-efficacy	High self-efficacy
Male	13	35	53
Female	16	43	41
Total	14	38	48

**Table 9:** Percentage of motor self-efficacy by school type.

School type	Low self-efficacy	Average self-efficacy	High self-efficacy
Fiscal	13	40	48
Fiscomisional	15	37	49
Private	16	38	47
Total	14	38	48

The gender difference in self-efficacy was statistically significant [*χ*^2^(2) = 96.85, *p* < 0.001, Cramer’s V = 0.13], indicating a small-to-moderate effect size, suggesting a consistent gap, albeit not one of great individual magnitude.

In contrast, differences by school type were small and not always significant, indicating relative stability in the self-efficacy profile across school types.

### 3.5 Objective 4: perceptions of students and teachers regarding motivations, barriers, and contextual conditions related to physical activity and screen time

The thematic analysis of the focus group interviews identified themes that help contextualize the quantitative findings. Among students, themes were organized around the meaning they attributed to physical activity, the main competitors for their active time, and their experience with PE. Among teachers, themes focused on the structural conditions of the school, pedagogical strategies regarding screen use and participation, and the understanding of sedentary behavior as a problem requiring continuity beyond the classroom. Tables 10–12 summarize the themes, subthemes, and their integration with quantitative findings through a joint display, allowing explicit identification of convergence, complementarity, and divergence between both strands.

**Table 10:** Thematic summary by students.

Theme	Sub-theme	Thematic evidence (discourse synthesis)	Interpretation and link to the study
Meanings of physical activity	Health and wellbeing	The practice is considered beneficial for feeling better, taking care of your health, and improving on a personal level.	Supports interpreting physical activity as a behavior with perceived value and personal meaning, consistent with the psychosocial approach.
Meanings of physical activity	Enjoyment and positive assessment	Physical activity is described as enjoyable when it includes a playful component.	Suggests a teaching opportunity: reinforcing positive experiences can promote adherence.
Barriers to practice	Screens in leisure	The use of devices and networks is identified as competing with the time available for travel.	Provides context for quantitative patterns in screen time, especially among older age groups.
Barriers to practice	Academic demands and time management	Daily schoolwork and responsibilities are mentioned as constraints to maintaining the practice outside the center.	Clarify the role of “available time” as a barrier relevant to interpreting changes over time or with age.
PE as an experience	Perceived difficulty and physical conditions	The difficulties in the subject are explained by the perceived difficulty of the exercises and by individual physical conditions.	It identifies perceived competence as a central factor for engagement in PE.
PE as an experience	Teacher and class evaluation	The narrative does not focus primarily on teacher rejection; instead, it shifts to the task and physical experience.	Avoid simplistic attributions and guide students to review task design, progression, and competency support.
Psychosocial resources involved	Autonomy and self-esteem	The speech contains references to feeling capable, making decisions, and maintaining personal confidence in practice.	Connect with the framework of self-determination and the role of self-efficacy in facilitating active behavior.

**Table 11:** Thematic summary by PE teachers.

Theme	Sub-theme	Thematic evidence (discourse synthesis)	Interpretation and link to the study
Terms and conditions of the center	Resources and infrastructure	Material limitations and perceived differences between schools are described, which determine what can be done in class.	Contextualizes differences by school type and suggests that the physical environment shapes opportunities for practice.
Structural constraints	Real-time class	It is proposed that planning be adjusted to the weekly time available per course.	Avoid overloading the PE classroom with changes without considering the time frame and curriculum.
Classroom management	Cell phone regulation	Measures are described to limit cell phone use during the session and reduce distractions.	Connect teaching practice with sedentary lifestyles and attention in class.
Teaching strategies	Engaging tasks and participation	The need to propose motivating and adapted activities to maintain student engagement is emphasized.	Align the intervention with motivational processes and the pedagogical climate.
Sedentary lifestyle and habits	Social problem and continuity	Sedentary lifestyles are understood as a broad phenomenon, with emphasis on continuity and the formation of habits outside the classroom.	Supports realistic recommendations: more coordinated school intervention with families and the community context.
PE and development	Link to wellbeing and performance	Connections between motivation, self-esteem, and aspects of school performance are mentioned.	It provides an interpretative framework consistent with the study’s objective and its psychosocial approach.

**Table 12:** Integration of quantitative and qualitative findings.

Quantitative finding	Qualitative theme	Type of integration	Meta-inference
High recreational screen time (29–39.45%)	“Screens compete with homework and leisure”	Convergence	Screen time operates as a structural competitor for active behaviors
Low self-efficacy in girls	“Girls feel less confident in PE”	Convergence/complementarity	Gender differences reflect socially shaped perceptions of competence and participation
Positive association between self-efficacy and physical activity (rho ≈ 0.05)	Students emphasize feeling capable as key for participation	Complementarity	Self-efficacy acts as a psychological mechanism linking context and behavior
Negative association between intrinsic motivation in PE and free physical activity	Students report barriers outside school (time, access, competing demands)	Divergence/explanation	Motivation in PE does not automatically transfer to leisure-time behavior due to contextual constraints
Differences by school context	Teachers highlight resources, infrastructure, and time limitations	Complementarity	Structural conditions shape opportunities for physical activity beyond individual motivation

The thematic convergence between students and teachers strengthens the interpretative credibility of the analysis; nonetheless, the qualitative findings should be understood as illustrative rather than generalizable, in line with the purposive sampling design.

#### 3.5.1 Students

In the students’ discourse, physical activity is mainly described as a behavior with personal meaning, associated with health, wellbeing, and improvement. At the same time, barriers arise from time management, especially due to competing demands between screens and homework (Table 10). In relation to PE, experiences suggest that difficulties in the subject are more closely associated with perceived task difficulty or individual physical conditions than with negative teacher assessments. Taken together, these findings reinforce the notion that the disposition towards practice may be favourable, but is conditioned by the organisation of leisure time and experiences of perceived competition.

#### 3.5.2 Physical education teachers

The teaching staff places the analysis within a more structural framework: resources, infrastructure, and actual class time shape the teaching approach and opportunities for practice. In addition, strategies are described for sustaining participation and regulating cell phone use during sessions, as well as for addressing perceptions of sedentary lifestyles as a social problem that requires consistent, stable habits outside school (Table 11). A joint display (Table 12) was used to explicitly link quantitative findings with qualitative themes, identifying patterns of convergence, complementarity, and divergence between both strands.

This perspective offers a structural interpretation of the observed differences between schools and, at the same time, suggests realistic avenues for intervention focused on the pedagogical climate, motivation, and screen management.

## 4 Discussion

This study provides associative evidence that, among adolescents enrolled in schools in Quito, physical activity and sedentary behavior are embedded within a framework in which daily habits, psychosocial resources, and school context converge. In particular, recreational screen time, with a notable weight of internet use, and sex differences in variables such as motor self-efficacy position PE as a key setting for understanding the psychological experience of physical activity and for guiding realistic intervention strategies to promote active lifestyles.

Although small, such effect sizes are commonly observed in behavioral epidemiology. Their potential relevance at the population level depends on both effect magnitude and exposure prevalence; however, the present study does not estimate population-level impact indicators (e.g., population attributable fractions), and therefore any interpretation beyond individual-level associations should be made with caution.

### 4.1 Sedentary behavior and screen time: an internet-centred pattern sensitive to age

The results show that non-compliance with the two-hour daily threshold is concentrated particularly in recreational internet use (29%; 95% CI: 27.80–30.20) and tends to increase with age. This profile is consistent with the shift observed during adolescence toward more connected and multifunctional forms of screen use, in which leisure, communication, and academic tasks intertwine, and sedentary time may become consolidated into routine. Recent literature supports the idea that screen time, especially when it increases steadily, is associated with less favorable indicators of mental health and adjustment, although effects are generally modest and sensitive to how exposure is defined and measured (Santos et al., 2023; Gao and Gao, 2024; Lu et al., 2024; Panjeti-Madan and Ranganathan, 2023). The two-hour cut-off should be understood as an operational benchmark for screening and surveillance rather than a universal risk threshold (Bull et al., 2020; Chaput et al., 2020; Canadian Society for Exercise Physiology, 2016). At the same time, recreational screen time is an indicator that captures only part of sedentary behavior, and emerging work stresses that different screen-based sedentary behaviors may vary in context, purpose, and cognitive engagement, which can translate into different associations with mental health (Zou et al., 2024; Zhang et al., 2025; Wang et al., 2025). Prospective evidence also links higher sedentary exposure to a greater risk of depressive symptoms in adolescents, while reinforcing that effect estimates depend on how sedentary behavior is operationalised (Huang et al., 2020).

These quantitative patterns are confirmed by the qualitative findings (Table 12), in which students explicitly describe screen use as competing with homework and leisure time, reinforcing the interpretation of screen time as a structural constraint on opportunities for physical activity.

In this context, the results of the present study should be interpreted as associative patterns rather than causal evidence, particularly given the cross-sectional design and the use of self-reported measures.

This interpretation gains further relevance when situated within integrated movement behavior frameworks. WHO guidelines and 24-h movement frameworks emphasize that the day represents a shared “time budget” among physical activity, sedentary behavior, and sleep; therefore, intervening in one component without considering the others often yields limited effects (Bull et al., 2020; Chaput et al., 2020; Canadian Society for Exercise Physiology, 2016). In this context, schools can play a preventive role by helping balance screen use with opportunities for physical activity, without reducing the issue to a moralizing message about screens (Pérez-Jorge et al., 2021). Empirical evidence in adolescents supports this integrated view, showing that combined profiles of physical activity, screen-based sedentary behavior, and sleep are associated with mental health indicators (Lu et al., 2021).

### 4.2 Physical activity and psychosocial factors and the complexity of motivational and competence-related processes

The associations observed between physical activity and psychosocial variables were statistically significant but small, a common pattern in large samples using self-report measures. Although statistically significant, the practical relevance of these associations should be interpreted cautiously given their small effect sizes.

In approximate terms, coefficients within the observed range imply low proportions of explained variance (often below 2%), reinforcing the idea that active behavior reflects a multifactorial configuration in which psychological resources operate as contributing rather than determining factors.

Nevertheless, the consistency of the link between self-efficacy and practice is relevant from both theoretical and applied perspectives. Self-efficacy, understood as the belief in one’s own capacity to organize and execute actions, is a central mechanism within the social-cognitive framework and has been repeatedly associated with the initiation and maintenance of active behaviors (Bandura, 1997). Moreover, evidence suggests that self-efficacy may mediate part of the effect of social support on physical activity in adolescents, aligning with models that combine personal resources and environmental conditions (Lin et al., 2024; Bauman et al., 2012). This aligns with adolescent evidence showing that self-efficacy can partly explain the link between perceived social support and physical activity (Ren et al., 2020), and with reviews indicating that self-efficacy and competence-related beliefs are among the most frequently examined mediators in youth physical activity interventions (Lubans et al., 2008). In addition, youth syntheses link physical activity to physical self-concept and perceived competence (Babic et al., 2014), and school-sport evidence suggests that beliefs and social support are intertwined with physical self-perception (Lubans et al., 2011).

From an applied perspective, even modest increases in self-efficacy may translate into cumulative changes in the frequency or duration of practice when sustained over time, reinforcing its value as a target for intervention in school contexts (Pérez-Jorge et al., 2024a,b). These findings are complemented by the qualitative results (Table 12), which highlight perceived competence as a key condition for participation, reinforcing the role of self-efficacy identified in the quantitative analysis.

By contrast, the inverse relationship observed between situational intrinsic motivation in PE and free physical activity requires careful interpretation. It does not imply that motivation is irrelevant; rather, it suggests that the transfer between motivational experience in class and behavior outside school is not automatic. Two plausible explanations, consistent with contemporary motivational frameworks, are: first, that a situational measure referring to the class may capture specific experiences related to a particular instructional unit or school moment rather than a stable disposition toward practice; second, that leisure-time behavior may depend on external barriers and facilitators (safety, access, family norms, available time) that may “dampen” the direct relationship between enjoyment in class and practice outside school.

Importantly, this counterintuitive pattern has been previously noted in the literature when examining the transfer of motivation across contexts. Transcontextual models suggest that motivation experienced within structured settings such as PE does not automatically translate into behavior outside the school context, particularly when opportunities, environmental constraints, and social support differ substantially (Hagger et al., 2009; Polet et al., 2021).

From a Self-Determination Theory perspective, intrinsic motivation in PE reflects a context-specific motivational state that may not generalize to leisure-time behavior if basic psychological needs are not similarly supported outside school (Ryan and Deci, 2017). In this sense, high enjoyment or engagement during PE lessons may coexist with low levels of autonomous physical activity if students face contextual barriers such as lack of access, time constraints, or competing sedentary alternatives.

Additionally, some studies have suggested that structured physical activity during school hours may partially compensate for or substitute free-time activity, particularly in adolescents with limited opportunities for extracurricular participation, which could contribute to inverse or weak associations between school-based motivation and leisure-time physical activity (Bauman et al., 2012).

Therefore, rather than indicating a negative role of intrinsic motivation, this finding may reflect the contextual specificity of motivational processes and the complex interplay between school experiences and out-of-school behavioral regulation. In this case, the qualitative findings extend the quantitative result (Table 12), suggesting that contextual barriers such as time constraints, competing academic demands, and limited access to opportunities may explain why motivation experienced in PE does not translate into leisure-time physical activity.

Likewise, the influence of unmeasured variables or reverse causality cannot be ruled out. Therefore, caution is warranted when interpreting the sign of the coefficient. This pattern may also reflect measurement specificity (situational versus habitual motivation), unmeasured constraints outside school, or statistical suppression when multiple psychosocial variables are considered simultaneously. It may also be partially influenced by measurement limitations inherent to self-report instruments.

This type of mismatch is compatible with transcontextual models and with Self-Determination Theory, in which teaching climate and satisfaction of basic psychological needs contribute to motivation, but its translation into behavior is moderated by opportunities and supports in other contexts. At the same time, competence-related goal orientations, as conceptualized within Achievement Goal Theory, may operate differently across contexts, contributing to variations in how motivational processes are expressed inside and outside the classroom (Deci and Ryan, 2000; Ryan and Deci, 2017; Standage et al., 2003; Polet et al., 2021).

This finding underscores the importance of analyzing not only motivational quality in the classroom but also the structural and social conditions that mediate the transfer toward autonomous practice.

These findings should also be interpreted considering that different motivational frameworks capture distinct dimensions of engagement. While goal orientations derived from Achievement Goal Theory reflect how students define competence and success (e.g., task vs. ego orientation), Self-Determination Theory variables reflect the quality and regulation of motivation along the autonomy continuum. This conceptual distinction may help explain the differential patterns observed across psychosocial and motivational variables, as they represent complementary rather than equivalent motivational processes.

### 4.3 Differences by gender and school context: a gap that involves perceived competence and opportunities

One of the findings with the greatest practical relevance is the sex difference in motor self-efficacy, with a less favorable profile observed among girls. From a conceptual standpoint, this gap may be linked to differentiated trajectories of motor experience and socialization, in which social comparison, body exposure, and performance expectations shape perceptions of competence and, consequently, willingness to participate. Evidence syntheses in youth indicate that physical activity is meaningfully related to physical self-concept and competence-related perceptions, highlighting why competence gaps can be consequential during adolescence (Babic et al., 2014). Moreover, school-sport research suggests that social support and beliefs about ability are closely connected to physical self-perception, reinforcing the importance of supportive climates for girls’ participation (Lubans et al., 2011). This pattern is confirmed and extended by the qualitative findings (Table 12), where girls explicitly report lower confidence and less favorable experiences in PE, suggesting that the observed gap is shaped by social and contextual factors. These differences can also be interpreted in light of gender socialization processes, which shape beliefs about physical competence and participation from early stages. Research has shown that girls are more likely to internalize lower perceptions of physical ability due to social norms, differential encouragement, and reduced opportunities for diverse motor experiences, particularly in contexts where physical competence is associated with traditionally masculine forms of performance.

In Physical Education settings, these dynamics may be reinforced or mitigated depending on the pedagogical climate. Studies suggest that performance-oriented environments and social comparison processes can disproportionately affect girls’ perceptions of competence, whereas autonomy-supportive and mastery-oriented climates may help reduce these gaps by fostering inclusive participation and emphasizing personal improvement over normative performance standards.

Therefore, the observed differences in motor self-efficacy should be understood not only as individual-level perceptions but as the result of broader sociocultural and educational processes that shape students’ experiences and opportunities for engagement in physical activity.

These differences should not be interpreted as fixed individual dispositions, but rather as the probable result of structural opportunities and accumulated experiences that shape perceptions of competence over time.

In PE, recent studies have highlighted the importance of perceived competence, enjoyment, and support for basic psychological needs as pathways to sustain engagement and participation (Guan et al., 2023; Kruse et al., 2024; Langøy et al., 2024). In addition, the relational dimension plays a role: the quality of the teacher–student relationship and peer acceptance have been linked to interest and participation in class, and may operate as protective or risk factors depending on the classroom climate (Chen et al., 2025; De Bruijn and Grimminger-Seidensticker, 2025).

Regarding differences by school type, the descriptive patterns suggest that institutional context matters, although interpretation should be cautious when the tables reflect group composition rather than prevalence within each school type.

Accordingly, these results should be understood as indications of differential patterns rather than as conclusive estimates of structural inequality across school types.

In any case, the literature on correlates of physical activity reminds us that opportunities, resources, environment, and access are key determinants, particularly when active behavior depends on material conditions and available provision (Bauman et al., 2012). The qualitative component, by emphasizing resources, infrastructure, and the actual organization of classes, proposes a plausible contextual mechanism that complements the quantitative interpretation.

### 4.4 Contribution of the qualitative component: what it explains and what it cannot guarantee

The thematic synthesis of the qualitative component reinforces two main ideas: students associate physical activity with health and wellbeing, but acknowledge competition from screen time and homework; teachers, for their part, frame motivation and classroom management in relation to structural limitations, resources, and infrastructure, as well as strategies to regulate mobile phone use during lessons. This convergence provides interpretative coherence and supports integrated meta-inferences across both strands, and connects with Self-Determination Theory, in which support for autonomy and competence, together with the quality of the relational bond, fosters more adaptive motivational experiences in PE (Deci and Ryan, 2000; Ryan and Deci, 2017; Kruse et al., 2024; Langøy et al., 2024; Wanwan and Khairani, 2025).

Despite this, the qualitative component does not allow the establishment of causal hierarchies among factors; rather, it proposes plausible interpretative mechanisms that enrich, but do not replace, the quantitative evidence.

The integration of quantitative and qualitative findings enables the development of meta-inferences that go beyond the contribution of each strand independently. While quantitative results identify small but consistent associations between psychosocial variables and physical activity, qualitative data reveal the contextual mechanisms that shape these relationships, including structural constraints, time competition with screen-based activities, and variations in perceived competence. Together, these findings suggest that adolescent physical activity is shaped not solely by individual motivation, but by the interaction between psychological resources and contextual opportunities. This integrated perspective highlights the importance of addressing both psychosocial and environmental factors in school-based interventions.

### 4.5 Implications

At the theoretical level, the results support an integrated interpretation in which self-efficacy and motivational processes should be conceptualized as complementary rather than competing processes, and in which the school context moderates experiences that may either facilitate or hinder active behavior. At the practical level, the implications focus on two fronts: reducing recreational sedentary time, particularly that associated with internet use, through realistic time-management and digital literacy strategies; and designing PE experiences that build perceived competence, autonomy, and enjoyment, with explicit attention to gender equity. At the methodological level, the study suggests that measurement and analysis benefit from combining behavioral indicators with psychosocial variables and interpreting them within a 24-h framework, rather than treating sedentary behavior as merely an “absence” of activity. Evidence from school-based interventions provides practical support for these directions, showing that multicomponent programs delivered through school settings can target both physical activity and screen-related behaviors within real-world constraints (Smith et al., 2014; Lubans et al., 2016a). Mechanism-focused syntheses further indicate that changes in self-perceptions, self-efficacy, and motivational processes are plausible pathways through which physical activity initiatives may influence broader cognitive and mental health outcomes in youth (Lubans et al., 2016b).

## 5 Limitations and future studies

The study has a large sample size and includes diverse schools and areas, allowing for a precise description of patterns and exploration of differences between subgroups. In addition, it integrates well-established instruments for psychosocial and behavioral variables and incorporates a qualitative component that enhances interpretation of the school context.

That said, the cross-sectional design prevents establishing causal relationships among psychosocial variables, sedentary behavior, and physical activity. Measurement is based on self-report, which entails a risk of recall bias and social desirability bias, particularly regarding minutes of activity and screen time. In the qualitative component, focus group data may also be subject to social desirability and group conformity biases. Adolescents may have adapted their responses to align with perceived peer norms or socially acceptable behaviors, especially when discussing sensitive topics such as screen time or physical inactivity. In addition, the group setting may have limited the expression of dissenting or less normative views, potentially influencing the depth and variability of the qualitative data.

Convenience sampling limits generalizability to the entire adolescent population of Quito and may introduce selection bias associated with institutional accessibility. In addition, schools that agreed to participate may have had more engaged administrations or a greater interest in physical activity promotion, potentially overrepresenting more favorable school climates. Furthermore, differences in school type may reflect underlying socioeconomic disparities, particularly in private and publicly funded private schools, which may limit the generalizability of the findings. Furthermore, interpreting differences by school type should be done with caution when results are presented as group composition rather than as prevalences within each category.

Future studies should employ longitudinal or intervention designs to explore underlying mechanisms, particularly those related to self-efficacy, psychological need satisfaction, and autonomy support in PE.

## 6 Conclusion

This study suggests that, among adolescents attending school in Quito, physical activity and sedentary behavior are better understood through an integrated approach that combines daily habits, psychosocial resources, and school contextual conditions. Recreational screen time—particularly internet use, which increases with age—emerges as a key behavioral factor to consider alongside efforts to promote physical activity. Motor self-efficacy shows a sex-based gradient and is associated with practice, supporting the relevance of school-based interventions that foster perceived competence, autonomy, and enjoyment in PE, with particular attention to gender equity. Overall, the findings provide useful evidence to guide school actions that simultaneously promote active lifestyles and reduce recreational sedentary behavior, tailored to the real conditions of educational settings.

## Data Availability

The raw data supporting the conclusions of this article will be made available by the authors, without undue reservation.
